# Management of Elevated Blood Pressure and Hypertension in Hospitalized Non-hypertensive Patients: A Systematic Guideline Review

**DOI:** 10.7759/cureus.85904

**Published:** 2025-06-13

**Authors:** Gift Ojukwu, Adanna N Ezike, Victor U Chukwu, Ogechukwu H Nnabude, Oghenemaro O Oghotuoma, Obianuju Nwauwa, Stella Ehi Egege, Chidiogo N Okafor, Cosmas O Ihezie, Okelue E Okobi, Chisom Mokwe

**Affiliations:** 1 General Practice, Leeds Teaching Hospitals NHS Trust, Leeds, GBR; 2 Geriatrics, Forth Valley Royal Hospital, Larbert, GBR; 3 College of Medicine, Abia State University, Uturu, NGA; 4 General Medicine, Windsor University School of Medicine, Cayon, KNA; 5 General Internal Medicine, Walsall Healthcare NHS Trust, Walsall, GBR; 6 Family Medicine, International University of the Health Sciences (IUHS), Calgary, CAN; 7 General Medicine, Enugu State University of Science and Technology, Enugu, NGA; 8 Orthopedics, Federal Teaching Hospital Owerri, Owerri, NGA; 9 Family Medicine, Larkin Community Hospital Palm Springs Campus, Hialeah, USA; 10 Family Medicine, Enugu State University Teaching Hospital Parklane, Enugu, NGA

**Keywords:** blood pressure, hypertension, inpatient, practice guidelines, transitional care

## Abstract

The management practices of elevated blood pressure (BP)/hypertension (HTN) in patients hospitalized for non-hypertensive reasons vary, despite such patients recording BPs much higher compared to the levels proposed for outpatient contexts. Moreover, despite elevated BP being a widespread dilemma faced by most healthcare practitioners (HCPs), there are no universal guidelines to aid with the management of elevated BP in patients hospitalized for non-HTN reasons. Therefore, this systematic review seeks to identify and evaluate the most recent and contemporary guidelines on managing elevated BP and HTN in hospitalized patients for non-hypertensive reasons.

To attain the stated objective, a robust method was employed to attain the study objective based on the Preferred Reporting Items for Systematic Reviews and Meta-Analyses (PRISMA) and Cochrane guidelines. Further, an exhaustive literature search was performed on online databases, including Medline, PubMed, Embase, Google Scholar, Web of Science, and Scopus. The study inclusion criteria stipulate that, to be included, the selected studies must have been published between 2015 and 2025, and in the English language. The selected studies were appraised using an appraisal tool for cross-sectional studies. Twelve studies satisfied the inclusion criteria and were reviewed.

The study has demonstrated that the lack of universal guidelines for elevated BP management for patients hospitalized for non-HTN reasons poses a challenge to many HCPs, despite the existence of significant literature on elevated BP management. Available guidelines have continued to focus on the management of elevated BP and HTN in outpatient contexts. Consequently, HCPs increasingly rely on outpatient BP management guidelines in treating hospitalized patients for non-HTN reasons. Thus, regardless of the existing universal consensus on outpatient BP management, at present, there is no general guidance on inpatient management of elevated BP in non-HTN patients, and this has significantly contributed to the observed variations in practice patterns.

## Introduction and background

Hypertension (HTN) remains a widespread chronic condition affecting United States adults, with a prevalence rate of nearly 50% in individuals aged 40-59 years, and nearly 70% prevalence rate for those aged 65 years and above [1,2]. Increasing age and HTN are strong risk factors for cardiovascular diseases (CVDs), affecting most older persons. In addition, the hospitalization rate of admission for inpatient and outpatient procedures is disproportionately higher [2]. Due to the higher HTN prevalence and its role as a key risk factor for CVD, established guidelines have been developed for the diagnosis and management of elevated blood pressure (BP) and HTN within outpatient contexts [3]. However, several hospitalized patients without HTN still present elevated BP and HTN by such standards. Regardless, this is reflective of HTN or temporary BP elevations resulting from severe illness or hospitalization. Existing studies have approximated that between 50% and 72% of inpatients often exhibit elevated BP levels [4].

Furthermore, it is notable that the management of elevated asymptomatic BP in patients hospitalized for non-hypertensive reasons remains highly variable, and significant inconsistencies have been reported in relation to medication routes, intensification of treatment, and novel antihypertensive prescriptions [4-8]. Currently, no evidence is available from randomized clinical trials that support intensive treatment of non-HTN patients, even as observational studies have indicated that approximately 34% of medical inpatients are offered intravenous BP medications, while nearly 14% of inpatients are discharged with intensified regimens following hospitalization for non-HTN reasons [6-8]. Additionally, the risks associated with treating asymptomatic elevated BP in non-HTN inpatients are still unclear due to the dearth of sufficient published clinical trials. Nonetheless, observational study data, despite having a high potential for confounding through indication, have associated intensive BP treatment in non-HTN inpatients with worse outcomes that include acute kidney injury (AKI), myocardial injury, and stroke [6-10]. Due to the increased variability in practice and the potential risks of over- and under-treatment, this study aims to review the existing BP management guidelines for patients hospitalized for non-HTN reasons, to identify recommendations for BP targets, treatment interventions, and post-discharge follow-ups.

Notably, in patients with elevated BP and HTN, various interventions have been acknowledged to minimize the incidence of cardiovascular conditions that include stroke, myocardial infarction, and heart failure, as well as all-cause and cardiovascular mortality. For instance, studies by Ettehad et al. and Thomopoulos et al. disclosed that a reduction of systolic BP by approximately 10 mm Hg in patients with elevated BP and HTN minimized the risk of the patient developing a major CVD event by nearly 20%, stroke by 27%, coronary heart disease by 17%, all-cause mortality by 13%, and heart failure by 28% [11,12]. Moreover, as noted earlier, a considerable proportion of hospitalized patients have undiagnosed HTN, even as individuals with diagnosed HTN usually are not offered antihypertensive drugs during their admission [13]. In many instances, elevated BP in hospitalized patients has been associated with pain, anxiety, and white-coat HTN, which might bring about spikes in BP, emphasizing the requirement for standardized protocols for BP measurements in hospitals to ascertain the collection of dependable data [13-16]. Medication adherence is vital as patients with difficulties adhering to outpatient medications are increasingly liable to experience elevated BP during hospitalization [13,14].

Additionally, BP treatment is often not initiated in many instances until the measurement exceeds 140/90 mmHg [13-16]. Various guidelines have stipulated that a clinical diagnosis of HTN should only be considered when the patient presents with severely elevated BP levels [9-11]. Moreover, follow-up referrals for discharged patients to determine the presence or absence of HTN remain inadequate. Inpatient HTN is frequently situational and transient, even though poorly managed elevated BP during hospitalization has been associated with adverse outcomes, including cardiac events and AKI, and unnecessary post-discharge antihypertensive medications [10,11]. Unfortunately, several studies have shown that patients hospitalized for non-HTN reasons and with elevated BPs always remain hypertensive after discharge and within their communities [2,12,15]. Patients hospitalized for non-HTN reasons but presenting elevated BPs have been associated with an increased risk of developing outpatient HTN at one month, as well as three years after discharge [16,17]. As such, inpatient admissions are perceived as offering essential opportunities for identifying both undiagnosed and uncontrolled HTN, as well as providing effective enhancements to post-discharge BP management. Furthermore, the recognition of inpatient elevated BP and HTN will likely provide the necessary opportunities for diagnosis and management intensification during hospital admission and in outpatient contexts. Nonetheless, identifying hospitalized patients with elevated BP and severe HTN remains a key challenge in clinical practice contexts due to the lack of universal diagnostic standards, as well as the effects of multiple BP measurements recorded in hospitals. This systematic review has also been necessitated by the lack of consensus on inpatient-specific BP targets, the significance and need for synthesized evidence on optimal interventions and strategies, and the risk for under- or overtreatment.

## Review

Materials and methods

Relevant research and peer-reviewed studies published in English between 2015 and 2025 were identified through an in-depth search of various online databases, including Scopus, PubMed, Web of Science, Google Scholar, and Embase. The articles chosen comprised epidemiological studies, multi-center studies, health assessment studies with de-identified data, and published review articles. Further, duplicate data sources were identified by comparing diverse articles and studies from comparable population years. For the literature search, we utilized Medical Subject Headings (MeSH) keywords, including HTN, BP, inpatients, hospitalization, and clinical practice guidelines. As a result, the search yielded a total of 326 references.

Inclusion and Exclusion Criteria

After removing the duplicates, pertinent guidelines were chosen using a three-stage process. The first phase involved screening the guidelines' titles and abstracts, while the second stage involved removing or excluding irrelevant guidelines. The third stage involved conducting a comprehensive review of the selected guidelines to validate their significance. Three independent reviewers screened the guidelines, and potential discrepancies were resolved through consultations and consensus.

Furthermore, the inclusion criteria specified that only original studies, such as randomized controlled trials (RCTs), crossover design studies, and prospective cohort studies, were to be included. The included studies were primarily scientific publications of original research, published in indexed and high-impact factor journals, between 2000 and 2025. As noted earlier, the studies also had to be published in English. To be included, the studies must have focused on managing elevated BP levels in non-hypertensive hospitalized patients. The exclusion criteria included guidelines that focused on the outpatient management of BP and guidelines that focused on the management of elevated BP levels in patients hospitalized for HTN-related reasons. Still, opinion pieces and editorials were excluded. Inaccessible guidelines and those with unsound methodologies were also excluded. The inclusion and exclusion criteria led to the exclusion of 314 references. Furthermore, for this study, relevant data were extracted from guidelines that met the inclusion criteria. The authors collected the attributes of the included guidelines alongside the findings.

Results

**Figure 1:**
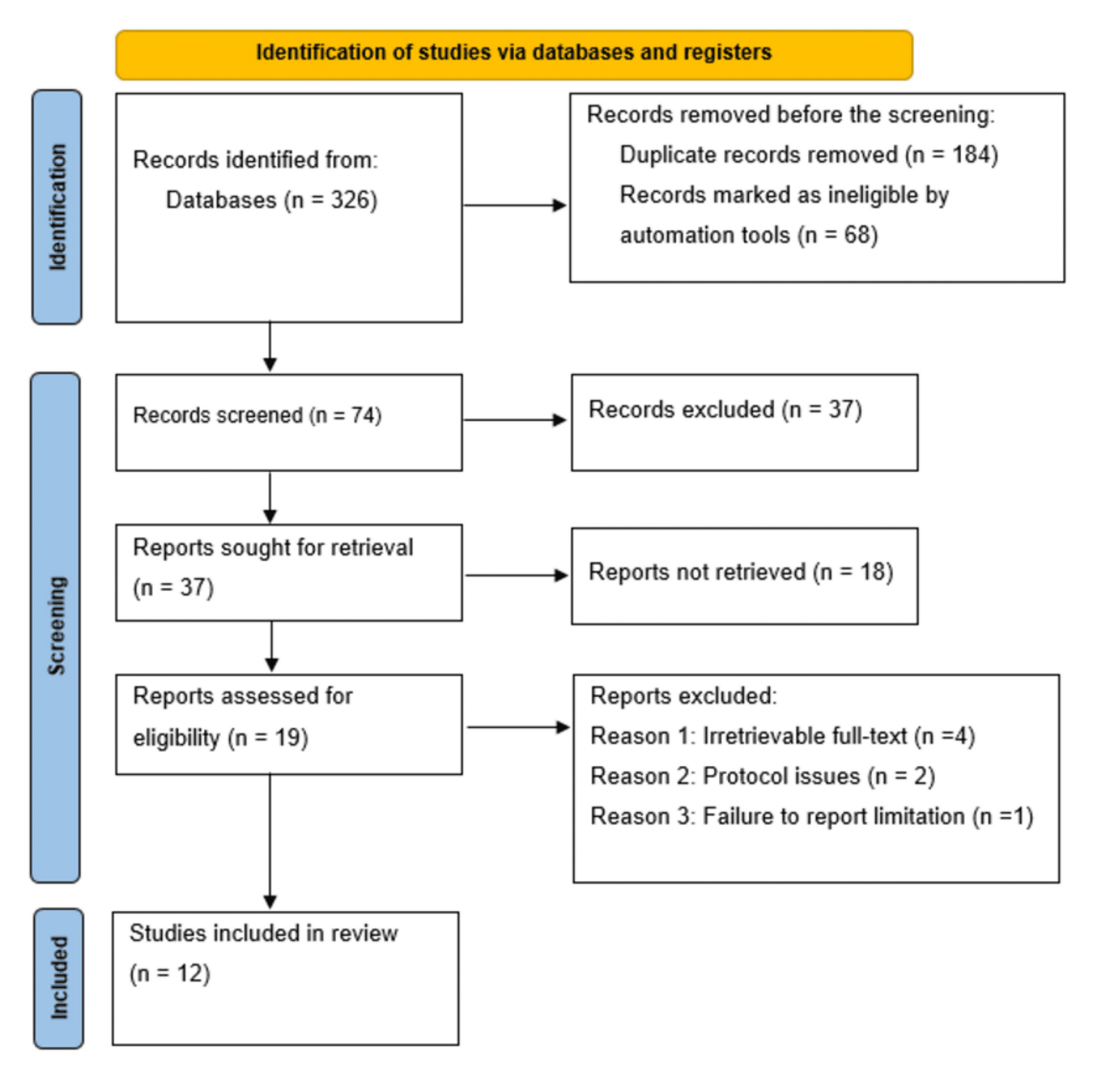
PRISMA flow diagram indicating the guideline selection process for this systematic review PRISMA: Preferred Reporting Items for Systematic Reviews and Meta-Analyses

For this systematic review, the guideline selection process was carried out using the Preferred Reporting Items for Systematic Reviews and Meta-Analyses (PRISMA) guidelines. Thus, a total of 326 unique references were screened to determine their eligibility for inclusion in the review. After screening, 184 duplicate guidelines and 68 articles found ineligible through automation were excluded. An additional 37 references were excluded for various reasons, including misalignment with the objective of this study. Ultimately, 37 eligible guidelines were screened, resulting in the exclusion of 18 guidelines. The remaining 19 guidelines were further sought for retrieval, with seven guidelines being irretrievable. As such, 12 guidelines were assessed for eligibility following full-text screening [17-28]. The PRISMA flow diagram in Figure 1 presents the guideline selection process.

Notably, the guidelines included in this study were drawn from different countries worldwide, and all of them provided broader management recommendations for elevated BP [17-28]. Additionally, two guidelines offered specific recommendations for managing elevated BP in elderly patients [17-24]. Moreover, three guidelines provided recommendations targeting specific clinical contexts, including recommendations for managing elevated asymptomatic BP within emergency department settings [21,22,26], with the other two offering recommendations specific to managing hypertensive urgencies or crises [18,20]. Consequently, two vital questions to effective management of patients with asymptomatic elevated BP within the emergency department settings, including whether screening for end-organ damage minimizes the risk of adverse outcomes and if medical interventions are prone to minimize the risk of adverse outcomes have been assessed by the guidelines developed by the American College of Emergency Physicians (ACEP), and have been found pertinent to management of elevated BP in inpatient contexts [29].

Quality Assessment of the Included Guidelines

The quality of the included guidelines was assessed using the Appraisal of Guidelines for Research and Evaluation II (AGREE II) quality assessment tool. Generally, the scores ranged between 3.5 and 6.5 out of a total score of 7.0. The scores have been indicated in Table 1. The guidelines developed by the U.S. Department of Veterans Affairs (VA) and the American College of Cardiology/American Heart Association (ACC/AHA) received the highest scores, with a rating of 6.5 [24,26]. The individual domain-scale scores range as follows: scope and purpose (60-100%), stakeholders’ involvement (34-97%), development rigor (32-98%), presentation clarity (88-100%), applicability (10-82%), and independence of the editorial (29-92%). Further, 10 guidelines were rated high quality based on the development rigor domain scaled score, given that their quality threshold was 68%. While eight guidelines had a systematic review background, the type of review employed by the other four was not explicitly stated. Nevertheless, none of the guidelines focused on managing elevated BP in patients hospitalized for non-hypertensive reasons, even as some guidelines addressed managing hypertensive urgencies and emergencies [18,23,29]. Despite the observed methodological limitations, data extraction from all guidelines enabled the capture of practical clinical practice recommendations.

**Table 1:** AGREE II quality assessment scores for the included guidelines ER: evidence review; SR: systematic review; AGREE II: Appraisal of Guidelines for Research and Evaluation II; AAP: American Academy of Pediatrics; ACC/AHA: American College of Cardiology/American Heart Association

Domain	AAP 2017 [17]	British and Irish Hypertension Society (BIHS) 2022 [18]	European Society of Hypertension (ESH) 2023 [19]	Hypertension Canada (HC) 2020 [20]	Japanese Society of Hypertension (JSH) 2019 [21]	National Institute for Health and Care Excellence (NICE) 2022 [22]	Qatari Ministry of Public Health (QMoH) 2021 [23]	Department of Veterans Affairs and Department of Defense (VA) 2020 [24]	World Health Organization (WHO) 2021 [25]	ACC/AHA 2017 [26]	Medscape 2024 [27]	ACC 2024 [28]
Evidence methods	ER	SR	ER	SR	SR	SR	SR	SR	SR	SR	ER	ER
Scope and purpose	60%	92%	64%	94%	100%	100%	100%	100%	100%	97%	100%	100%
Stakeholder involvement	34%	42%	67%	78%	64%	97%	83%	94%	69%	92%	72%	97%
Rigor of development	32%	32%	60%	82%	68%	85%	78%	98%	88%	97%	82%	85%
Presentation clarity	94%	89%	100%	100%	94%	97%	100%	100%	100%	100%	88%	97%
Applicability	63%	38%	42%	63%	29%	69%	35%	82%	79%	75%	10%	69%
Independence of editorial	56%	29%	42%	79%	33%	92%	50%	92%	100%	83%	68%	92%
Overall score (1-7)	5.5	4.5	5.5	6	4.5	6	5.5	6.5	6	6.5	6%	6
Quality level	High	Low	High	High	Low	High	High	High	High	High	High	High
Recommended this guideline for use?												
Yes	0	2	0	2	0	2	0	2	2	2	0	2
Yes, with modifications	2	0	2	0	2	0	2	0	0	0	2	0
No	0	0	0	0	0	0	0	0	0	0	0	0

Comprehensive Synthesis and Analysis of Included Guidelines

The review has disclosed that all guidelines recommended comparable practices regarding the management of elevated BP within outpatient contexts and the treatment of hypertensive emergencies, even though there were no recommendations regarding the management of elevated asymptomatic BP in patients hospitalized for non-hypertensive reasons [17-28]. Furthermore, regarding the management of hypertensive emergencies, five of the reviewed guidelines provided recommendations for managing severe BP elevations in inpatients, classified as hypertensive emergencies and urgencies [19,21-23,26]. Aspects including BP elevation above threshold and end organ damage have been consistently defined as hypertensive crises, mostly >180/120 mm Hg [19,21-23,25,26,28]. Additionally, the guidelines proposed treatment for elevated BP in inpatient contexts, mainly in the intensive care unit (ICU), using intravenous medications [21-23,26]. Despite offering detailed management recommendations on definite elevated BP-related emergencies, the identified emergencies significantly differed by emergency/crisis type, including an instant BP reduction for aortic dissections [19,21-23,26], as well as a gradual decrement of BP over days in instances of acute renal failure and malignant HTN [19,21,26]. Nevertheless, all 12 guidelines reviewed specified hypertensive encephalopathy and severe coronary syndrome [17-28]. Still, of the reviewed guidelines that specified hypertensive encephalopathy, only five provided proposals for management, and four offered proposals of a 25% BP reduction over two hours [18,19,23,26-28].

Many guidelines have not provided definitive recommendations for effectively managing asymptomatic elevated BP in inpatient settings, such as the preferred antihypertensive medication classes and target BP levels for hospitalized patients. For instance, a total of nine guidelines tackled the challenge of hypertensive urgencies, characteristically defined by BP levels of >180/120 mm Hg devoid of any end-organ damage; nevertheless, the guidelines mostly concentrated on management within the emergency and outpatient contexts, with minimal focus on inpatient management and care [18,19,21-23,25-28]. Therefore, most guidelines have proposed outpatient management of elevated BP using limited immediate interventions and oral medications. Inconsistent proposals for diagnostic assessment of end-organ damage can also be observed across the guidelines, with only five guidelines recommending the performance of a definitive test based on the patient’s symptoms [18,21-23,28].

Due to the lack of guidelines for managing elevated BP in patients hospitalized for non-high BP (HTN) reasons, healthcare providers are increasingly likely to rely on outpatient guidelines, as defined in the reviewed guidelines. Typically, the targets for outpatient BP range between <130/80 mm Hg and <140/90 mm Hg, even as the thresholds for pharmacological interventions have been defined as between >140/90 mm Hg and >160/100 mm Hg [19,23,26,28]. The use of monotherapy with angiotensin-converting enzyme (ACE) inhibitors, angiotensin II receptor blockers (ARBs), thiazides, and calcium channel blockers (CCBs), as well as combination treatment for individuals with stage 2 HTN, has been recommended [20,23,24,28]. Notably, the outpatient guideline proposals have additionally accounted for the patient-definite factors, including comorbidities (heart failure, diabetes, and kidney disease), alongside geriatric considerations that include dementia and frailty, thereby calling for tailored treatment goals to aid in minimizing the side effects and maintaining their quality of life [19,23,24,26-28].

Furthermore, regarding care transitions, the evaluated guidelines have not provided recommendations for effectively managing elevated BP during patient transitions from hospital to their homes or communities, even after a hypertensive emergency. The only guidelines offered for transition of care have focused on the patient's transition from the emergency department to the outpatient contexts, and seven guidelines have aptly tackled this [18-23,26]. Thus, four guidelines have recommended that follow-ups should be conducted within seven days after discharge [19,21,22,24], and one guideline recommended that follow-ups should be performed within three days after discharge [28]. Two guidelines did not offer specific follow-up timelines [20,25]. Moreover, follow-ups for elevated BPs have been recommended and subsequently included for outpatient care, indicating that follow-up should be conducted within 30 days for moderately elevated BP and within one week for individuals with severely elevated BPs [20,25,26].

Discussion

Elevated BP management within the inpatient context remains a key challenge, even as approximately 50% to 70% of adult patients admitted to hospitals tend to experience BP elevations of ≥140/90 mm Hg during admission [30,31]. The BP elevation is attributable to underlying essential HTN, imprecise measurements, physiological stressors related to severe illness and hospitalization, and iatrogenic causes [6]. Chronic asymptomatic HTN has been defined as a severely elevated BP (>180/120 mm Hg) in the absence of severe HTN-mediated and targeted organ damage [4,6]. Regardless of the availability of evidence-based guidelines for managing outpatient elevated BP and HTN, a study conducted by Whelton and Williams disclosed that there were no clinical practice guidelines for managing elevated BP in patients hospitalized for non-HTN reasons within the inpatient contexts [32].

Despite being widely regarded as an outpatient condition, elevated BP is recurrently reported and managed within hospital contexts in which the optimal diagnosis and management practices vary and are uncertain. In their study, Penmatsa et al. (2021) noted that the inpatient elevated BP and HTN prevalence was between 50% and 71% [33]. Nevertheless, such a proportion probably underrates the prevalence, given that a significant percentage of patients have elevated BP levels that are either undetected or obscured, lest a 24-hour BP monitoring is carried out during hospital admission. Moreover, according to Conen et al., it is estimated that nearly 38% of all hospitalized individuals reporting elevated BP do not have any earlier HTN diagnosis [34].

Consequently, significant differences have been reported between existing HTN management guidelines regarding when to initiate pharmacological treatment for elevated BP in inpatients [26,30,31]. For instance, existing American guidelines have classified elevated BP of >130/80 mm Hg as HTN and recommended the initiation of pharmacological treatment for BP levels that exceed the threshold, particularly if BP is >20/10 mm Hg above the goal [26]. On the other hand, Canadian guidelines have recommended the initiation of pharmacological treatment when the BP level is ≥160/100 mm Hg in individuals considered low-risk and a BP level of ≥140/90 mm Hg in individuals with target organ damage and cardiovascular risk factors, with a treatment objective of <140/90 mm Hg [31]. Still, the European guidelines have recommended the initiation of pharmacological treatment of elevated BP at ≥140/90 mm Hg to realize a target systolic BP of 120-129 mm Hg in individuals below 65 years of age and 130-139 mm Hg in individuals 65 years and older [30]. Contrary to these recommendations, recent evidence has indicated that hospital-admitted and asymptomatic patients presenting with elevated BP face a high risk of adverse outcomes with the use of parenteral antihypertensive medications and up-titration of oral medications during admission [16]. Though the initial clinical practice guidelines for the management of HTN did not have such guidance for inpatients, the AHA has recently issued its initial recommendations for elevated BP management within the inpatient contexts, integrating the most pertinent and recent data [16].

Moreover, various guidelines for elevated BP and HTN management have stipulated that, within the outpatient contexts, HTN is defined through the average systolic BP ≥ 130 mm Hg or diastolic BP ≥ 80 mm Hg [26,30]. In agreement, Axon et al. maintained that inconsistencies exist about the definition and nomenclature of elevated BP in hospitalized patients without HTN [4]. Additionally, no precise BP readings stipulate what constitutes a hypertensive crisis, even though one guideline has reported that BP measurement of >179/109 mmHg is considered an appropriate threshold [3]. In this regard, the ACC/AHA guidelines recommend using laboratory assessments for patients with new elevated BP or HTN diagnosis to facilitate risk factor profiling, establish the baseline for medication use, and screening for secondary causes of the elevated BP [26].

Additionally, this systematic review of the various selected guidelines on elevated BP management in patients hospitalized for non-HTN reasons in this systematic review has disclosed that no guideline offered recommendations for use in the management of asymptomatic elevated BP within the inpatient care contexts. Notably, the recommendations on inpatient care have mainly focused on hypertensive emergency management without discussions regarding transitional management of elevated BP following discharge from the hospital. Contrary to the limited availability of guidelines focusing on elevated BP management within inpatient contexts, the included guidelines in this study have consistently outlined BP targets for outpatient management contexts, preferred classes of medication, and treatment thresholds, in addition to making recommendations for follow-up intervals and customized interventions for patients with certain geriatric conditions and comorbidities [17-28]. As such, the observed lack of comparable recommendations for use in managing elevated BP in inpatient care might contribute to the significant variability in clinical practices [4-8,10].

Owing to the lack of guidelines, various observational trials pertaining to the inpatient management of elevated BP have disclosed significantly dissimilar antihypertensive treatment patterns in both inpatient care and at discharge, including the usage of intravenous medications for moderately elevated BPs, medication intensification, and novel long-term antihypertensive initiation [4,6-8]. Several observational studies have further disclosed that increasingly intensive management of elevated BP in hospitalized patients is not linked to reduction of the cardiovascular outcomes in hospitalized patients, as well as that intensification of hypertensives with discharge is not related to reductions in cardiovascular outcomes [4,6,7]. Even though such studies are likely to be influenced by unmeasured confounding and selection bias, they make up the existing evidence body, owing to the lack of randomized trials to inform guidelines on inpatient elevated BP management.

Despite being highly detailed and significant, given the conditions’ potential to become life-threatening, the existing guidelines and recommendations for the management of hypertensive emergencies are inadequate, owing to their 0.3% prevalence rate of hospitalizations, even as asymptomatic elevated BP levels occur in most inpatient cases [23,22,28]. Further, the BP thresholds for establishing severely elevated BP levels have also differed across existing guidelines [20,25,26] and have been defined mainly by expert opinions and consensus as opposed to findings of clinical trials. Due to chronically elevated BP levels, emergencies and urgencies have been summed up jointly in existing guidelines, which might mislead. Thus, though emergencies normally require immediate treatments and, if left untreated, might result in either morbidity or mortality, the existing guidelines are in agreement that hypertensive urgencies are not urgent and have recommended treatment and follow-ups within days following presentation and not immediately [17,22,27,28].

Furthermore, the guidelines differed notably regarding the importance of testing for end-organ damage in cases involving severe BP elevations, given that BP elevation alone is not predictive of end-organ damage [19,25]. Also, a more significant proportion of the reviewed guidelines did not offer definite recommendations regarding measuring inpatient BP to verify and determine severe BP elevations effectively. Such a lack of diagnostic guidelines is likely to result in either the underuse of testing in instances where the patient is unable to effectively communicate the symptoms or the overuse of testing through the evaluation of all patients for end-organ damage [4]. As such, further clinical trials are required to establish the testing of end-organ damage’s diagnostic value, particularly in hospitalized patients with elevated BPs, comparable to the progress realized in the assessment of syncope diagnostic approaches, which is an intricate and severe condition indicative of serious pathology that is also self-limiting, with increasingly variable clinical testing practices [17,21,26].

Still, given the lack of guidelines stipulating targets for inpatient BP, physicians normally depend on recommendations for outpatient BP management. The outpatient guidelines have been adopted widely due to their in-depth and consistent advice on treatment thresholds, medication choices, and BP targets. Nonetheless, outpatient recommendations have been designed primarily for stable patients with the objective of reducing long-term cardiovascular risk and might not be applicable in cases involving acute illnesses [17,20]. Transient BP elevations, comparable to physiological blood glucose and heart rate increments during severe stress, might mark such cases. Treating elevated BPs without properly considering their temporary nature might result in unintended hypotension during hospitalization or upon the patient resuming the baseline physiological state [17,19,27,28]. Moreover, frequent BP monitoring within the inpatient contexts might result in adjustments in the management within hours in instances where instant BP reductions are not realized, despite long-acting antihypertensives normally needing a maximum of two weeks to realize a steady state. The intervention contradicts the recommendations for outpatient BP elevation management, stipulating follow-ups for weeks and months to effectively assess the effects of antihypertensive medication adjustments [19,26,27]. As a result, a higher risk of premature intensification of the treatment regimen exists, especially in hospitalized patients, which might additionally predispose the inpatients to hypotension and other adverse effects. Devoid of symptomatic hypotension, unnecessary overtreatments might expose the inpatients to medication-associated harms without any adequate opportunities for clinical benefits.

Still, care transition after hospital discharge also represents a significant area that the guidelines have inadequately tackled. No guideline has provided recommendations pertaining to medication reconciliation, discharge education, and the execution of home BP monitoring. It is unclear the percentage of patients advised to monitor their BPs at home following hospital discharge and those offered the essential monitoring equipment [15,16]. The increasing prevalence of home BP monitoring within outpatient care contexts might have significant roles post-hospitalization [33]. A few guidelines reviewed have provided recommendations for post-discharge follow-ups following hypertensive urgencies, proposing that follow-ups should be conducted within two months in instances of moderately elevated BP levels and within one week in cases of severely elevated BP levels [17,28]. Timely follow-up post-hospitalization might benefit patients discharged with novel antihypertensive medications to assess safety and efficacy, and patients with unchanged medication regimens to evaluate ongoing BP control [17,27,28]. However, more patients are not offered follow-up care within a timely window after hospital discharge [19,20,22,28]. Possible underlying reasons for the lack of follow-up include the barriers to accessing appointments, including challenges in securing a clinic visit within the stipulated timelines as a result of high demand, scheduling delays, and limited provider availability. Additionally, patient-level challenges include financial constraints and a lack of awareness about the significance of post-discharge follow-up, as well as inadequate communication between the patients and the outpatient teams that leads to missed follow-ups [19,20,22,28].

Owing to the limited number of observational studies focusing on managing inpatient elevated BP, practical clinical trials are required to assess the benefits and risks of different treatment interventions. Comparable to studies that focused on inpatient diabetes care, prospective studies should conduct comparisons on different thresholds for treating elevated BP levels in hospitalized patients. Despite the possibility that such trials are likely to be costly and require bigger sample sizes, owing to the infrequency of cardiovascular events in patients hospitalized for non-cardiac reasons, the studies are justified by the increased prevalence rate of elevated BP levels in hospitalized patients. Moreover, the proper definition of BP targets in hospitalized patients has remained a challenge, which has been attributed to the increasingly dynamic changes resulting from severe disease [19,21,22]. In this regard, an appropriate comparison for BP in hospitalized patients might be the heart rate, which, similar to BP, normally rises due to stressors that include dehydration and pain, but does not require routine treatment except in symptomatic instances. Rather, the characteristic management of tachycardia occurs through the tackling of various underlying causes, an intervention that might be applicable to the management of elevated BP in patients hospitalized for non-HTN reasons. Owing to the distinctive context of hospitalization and care transition, effective provisional guidelines are necessary to support clinical decision-making until comprehensive clinical trials are carried out. Thus, inpatient care services providers need customized care frameworks instead of dependence on outpatient guidelines targeted at divergent patient populations with distinct care timelines [17,20,24,26].

Strengths and Limitations of the Study

This systematic review has a number of limitations. For instance, despite the pre-registration of the search strategies, no peer review was conducted. Additionally, our evidence base has been limited to only guidelines published in English, with a restriction to guidelines published between 2010 and 2025. This likely resulted in the exclusion of several pertinent published guidelines. Regardless of using pre-defined criteria and several strategies, the dependence on MeSH terms in identifying pertinent guidelines is perceived as a limitation, given that it might have omitted various relevant and applicable guidelines. Definite inpatient clinical scenarios, including perioperative management and pregnancy, have been excluded from this systematic review owing to their increasingly distinctive evidence base. Moreover, for this systematic review, increased focus was placed on the contents of the guidelines, devoid of assessment of the underlying evidence that supports the various recommendations. Lastly, the study selection criteria also excluded the non-guideline sources, including narrative reviews and expert opinions regarding managing elevated BP within the inpatient contexts.

## Conclusions

In conclusion, elevated BP in patients hospitalized for non-hypertensive reasons remains a widespread yet poorly managed clinical issue that affects a significant portion of inpatients. Despite the existence of well-defined outpatient HTN guidelines, evidence-based standards for inpatient management are still lacking, leading to inconsistent practices. Existing recommendations differ widely with regard to treatment thresholds, handling asymptomatic HTN, and target BP levels, regularly depending on expert opinion as opposed to strong clinical data. Notable gaps include the lack of standardized diagnostic protocols, insufficient transitional care interventions and strategies, and unclear guidelines for assessment of end-organ damage. Thus, various studies have suggested that aggressive BP lowering is ineffective in improving outcomes and might even result in harm, including iatrogenic hypotension, given the potential of acute illness to cause transient BP spikes that might not need intervention. Owing to the higher prevalence of elevated inpatient BP alongside the risks of inappropriate treatment, there is an urgent need for RCTs to establish evidence-based strategies. However, until this is realized, the various provisional guidelines should tackle issues regarding diagnostic uncertainty, care transitions, and treatment thresholds, even as clinicians should prioritize personalized and careful management over reflexive antihypertensive intensification. This recommended approach will balance the potential benefits against the perceived risks of overtreatment in vulnerable individuals.
